# Outcomes of endovascular treatment of renal arterial stenosis in transplanted kidneys

**DOI:** 10.1590/S1677-5538.IBJU.2018.0737

**Published:** 2019-01-29

**Authors:** Giovanni Scala Marchini, Alexandre Sallum Bull, Affonso Celso Piovesan, Kleiton Gabriel Ribeiro Yamaçake, Ioannis Michel Antonopoulos, Renato Falci, Hideki Kanashiro, Gustavo Ebaid, Francisco César Carnevale, Gustavo Messi, William Carlos Nahas

**Affiliations:** 1 Unidade de Transplante Renal Faculdade de Medicina Universidade de São Paulo São Paulo SP Brasil Divisão de Urologia, Unidade de Transplante Renal, Faculdade de Medicina da Universidade de São Paulo - USP, São Paulo, SP, Brasil;; 2 Unidade de Radiologia Intervencionista Faculdade de Medicina Universidade de São Paulo São Paulo SP Brasil Unidade de Radiologia Intervencionista da Faculdade de Medicina da Universidade de São Paulo - USP, São Paulo, SP, Brasil

**Keywords:** Transplantation, Kidney, Arteries

## Abstract

**Objective:**

To evaluate the effectiveness and outcomes of endovascular treatment of TRAS with PTA.

**Materials and Methods:**

We searched our prospectively collected database looking at cases of TRAS between January 2005-December 2011. CCT was the gold-standart for diagnosis of TRAS. Parameters analysed comprised technical aspects, arterial blood pressure variation, and renal function. A minimum follow-up of 24 months was considered.

**Results:**

Of the 2221 renal transplants performed in the selected period, 22 (0.9%) patients were identified with TRAS. Fourteen (63.6%) were male and mean age was 377±14.8years (12-69). Kidney graft was from deceased donnors in 20 (80%) cases. On doppler evaluation, mean blood flow speed after transplantation, at TRAS diagnosis and after TAP was 210.6±99.5, 417±122.7 and 182.5±81.6mL/sec, respectively (p<0.001). For SBP and DBP, there was a significant difference between between pre-intervention and all post-treatment time points (p<0.001). After 1 month of the procedure, there was stabilization of the Cr level with a significant difference between mean Cr levels along time (p<0.001). After a mean follow-up of 16±4.2 (3-24) months, overall success rate was 100%.

**Conclusions:**

Endovascular treatment with PTA/stenting is a safe and effective option for managing TRAS, ensuring the functionality of the graft and normalization of blood pressure and renal function.

## INTRODUCTION

Renal transplantation remains the gold-standart treatment for patients with chronic renal failure, not only for a medical stand-point but also for an economic and social perspective. Indications include: end-stage renal disease (ESRD), dyalitic patients and in a preenptive matter.

The most common vascular complication in transplanted kidneys is transplant renal artery stenosis (TRAS), with a reported incidence of 1% in selected cohorts. However, literature incidence of TRAS is heterogeneous and usually higher, affecting almost 25% of patients according to some series (1-5). Therefore, appropriate diagnosis and treatment is crutial for organ preservation. TRAS usually occurs within the first three years after transplantation and its main cause is artheriosclerosis (6, 7).

Percutaneous transluminal angioplasty (PTA) is considered the less invasive approach for initial management of TRAS with proved efficacy (8). The purpose of our investigation was to evaluate the effectiveness of endovascular treatment of TRAS with PTA in regards to clinical and renal functional parameters, also comparing the results according to the site and etiology of TRAS.

## MATERIALS AND METHODS

### Case Selection

After Institutional Ethical Approval, a retrospective search on our prospectively collected database was performed searching for cases of TRAS between January 2005 and December 2011. TRAS was suspected if patient had refractory hypertension and transplant graft dysfunction, or when imaging exams were strongly suggestive of the disease. Initial evaluation was performed with doppler ultrassound and confirmed with contrasted computed tomography (CCT) with arterial phase reconstruction. Only cases confirmed by CCT were considered to have definitive diagnosis of TRAS. Of the 2221 renal transplants performed at our Institution in the selected period, 22 (0.9%) patients were identified with TRAS and all cases were treated with PTA.

### Surgical Technique

Initial angiography was performed to evaluate stenosis under ipsilateral common femoral artery approach with a 6-French guiding catheter using a nonionic isosmolar contrast medium. After a intravenous bolus of 5000UI of heparin a 0.014 inches guidewire was used to cross the stenosis and was positioned into a distal renal branch. A 6 x 15mm metallic balloon-expandable Palmaz Blue^®^ stent (Cordis, Johnson & Johnson) was positioned and primary stent placement was performed under roadmap technique. Arteriography showed ideal stent positioning without residual stenosis or complications. An Exoseal® closure device (Cordis, Johnson & Johnson) was used for hemostasis.

### Analysed Parameters

Analysed parameters comprised technical aspects, arterial blood pressure variation-systolic and dyastolic; need for antihypertensive drugs; and renal function-serum creatinine (Cr) variation. Blood pressure measurements considered the month before PTA, 2 days, 1 week, 1 month, 6 months, 1 and 2 years after the intervention. Cr values were obtained one week before intervention, 1 day, 3 days, 1 week, 1 month, 6 months, 1 and 2 years after PTA. Blood flow speed at doppler ultrassound evaluation was also obtained after transplantation, at TRAS diagnosis, and after angioplasty.

A subgroup analysis was performed to seek for differences regarding the above-mentioned parameters between groups divided by specific cause of TRAS and donnor characteristics. A minimum follow-up of 24 months was considered for all cases. Final success of PTA was considered if the procedure underwent uneventfull and if there was improvement of renal function paralell to blood pressure normalization.

### Statistical analysis

Data was analyzed with SPSS V.20 (Chicago, IL). Results were expressed in mean±standard variation and range, or absolute number and frequencies. The Chi-Square or Fisher Exact Test were used to compare categorical variales. To compare measurements for each parameter between individuals the Levene test was used. One-Way Analysis of Variance (ANOVA) with repeated measures was used to compare continuous variables among different time periods for each patient. ANOVA with fixed factor was used to compare continuous variables among groups. Post-Hoc test was performed with Tukey test. Statistical siginificance was set at p <0.05 and considered a 95% Confidence Interval.

## RESULTS

### Demographic Data

In the six-year period analyzed, 22 consecutive patients presented with TRAS after kidney transplantation-TRAS incidence of 0.9%. Of those, 14 (63.6%) were male and 8 (46.4%) female. Mean age was 37.7±14.8 (12-69) years.

Kidney graft was from deceased donnors in 20 (80%) cases and from living donors in 5 (20%). All presented with refractory hypertension and/or increased serum creatinine levels. The mean interval time from transplantation to diagnosis was 145 day±111 (4 to 232) days.

In 6 (27.3%) patients the site of renal artery stenosis was at the anastomosis. In 3 (13.6%) cases it was due to kinking and the remaining 13 (59.1%) cases it was distal to the anastomosis.

### Imaging Evaluation

**Figure 1:**
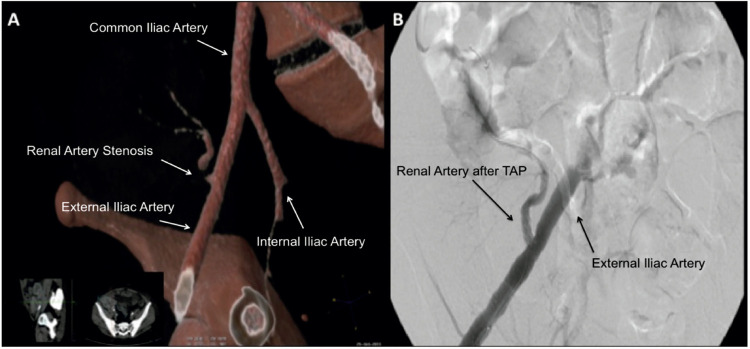
Three-dimensional reconstruction of arteriography showing TRAS due to kinking (A). Arteriography evidenciating successful outcome after stent placement at the kinking site (B).

On doppler evaluation, mean blood flow speed after transplantation, at TRAS diagnosis, and after TAP was 210.6±99.5, 417±122.7 and 182.5±81.6mL/sec, respectively (p <0.001). There was a significant difference between mean blood flow speed at time of TRAS diagnosis compared to after transplantation and after angioplasty, these two moments being similar. For each time point, there was no mean blood flow speed difference among patients (p=0.381), showing a homogeneous normalization of the speed after angioplasty. Figure-1 illustrates a case of TRAS due to kinking (Figure-1A) which was successfully treated with stenting (Figure-1B).

### Blood Pressure Analysis

**Figure 2:**
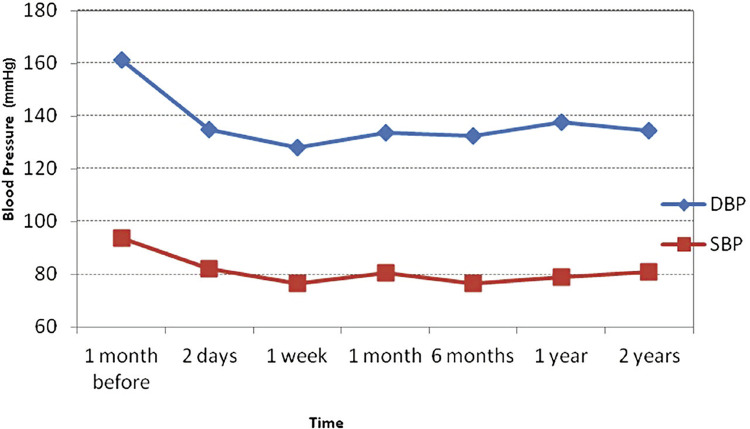
Systolic and diastolic blood pressure curves before and after TRAS treatment with TAP.

Mean diastolic blood pressure (DBP) showed a mean decrease of 23.4-33.0mmHg from pre-treatment to after intervention. Mean systolic blood pressure (SBP) also presented an average decrease of 11.8 to 17.3mmHg in the same period. After PTA, both showed a relative stabilization (Figure-2).

At each time point, there was no statistical significant difference among patients for SBP and DBP (p=0.11). For SBP and DBP, there was a significant difference between mean pressure value along the time period analysed (p <0.001) due to a significant difference between pre-intervention and all post-treatment time points (p <0.001). After the procedure, mean pressure value was similar for all patients at all time points (p non-significant).

Finally, when considering the amount of use of antihypertensive drugs, there was no statistically significant reduction in the number of medications used to control the disease.

### Renal Function Evaluation

Mean serum Cr level pre-intervention was 4.07±2.82mg/dL, falling to 2.1±1.94 after 2 years of TAP. At each time point, there was no significant mean Cr level between patients.

**Figure 3:**
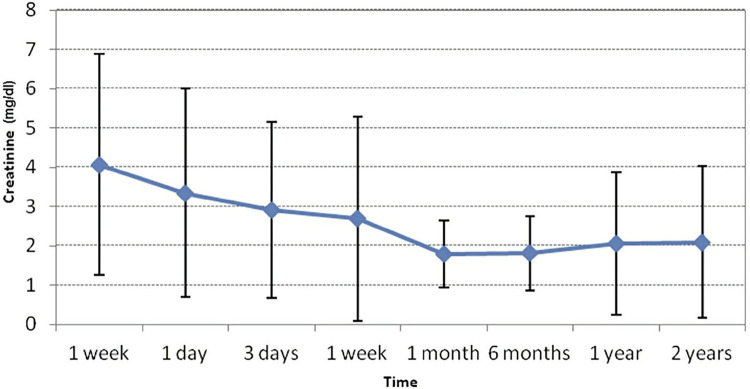
Curve progress of creatinine levels after PTA/stenting.

The mean drop of creatinine values from pre-intervention to post-treatment days 1, 3, 7 and 30 was 0.72, 1.16, 1.38 and 2.28mg/dL, respectively. After 1 month of the procedure, there was stabilization of the Cr level (Figure-3). Overall, there was a significant difference between mean Cr levels along time (p <0.001). Although there is a pronounced decrease in mean Cr level from pre to post-intervention, it turned statisticaly significant after the first month of TAP (p <0.01).

### Outcome Analysis

After a mean follow-up of 16±4.2 (3-24) months, overall success rate was 100%. No complications neither restenosis occured. Six patients died due to unrelated causes: 3 due to pneumonia, 1 due to vascular cerebral accident, 1 gastric tumor and 1 acute myocardial infarction.

### Stenotic Site Analysis

Age was found to be significantly different between groups (p=0.018). The kinking cohort (30.5±20.4 years old) was significantly younger than the anastomotic (50.3±10.2 years old) and post-anastomotic groups (56.2±6.2 years old)(p=0.002).

There was no difference for SBP between groups (p=0.34). However, DBP was different among cohorts (p=0.036). DBP was significantly lower in the kinking group compared to the anastomosis (p=0.029) but not to the post-anastomotic group (p=0.11).

**Figure 4:**
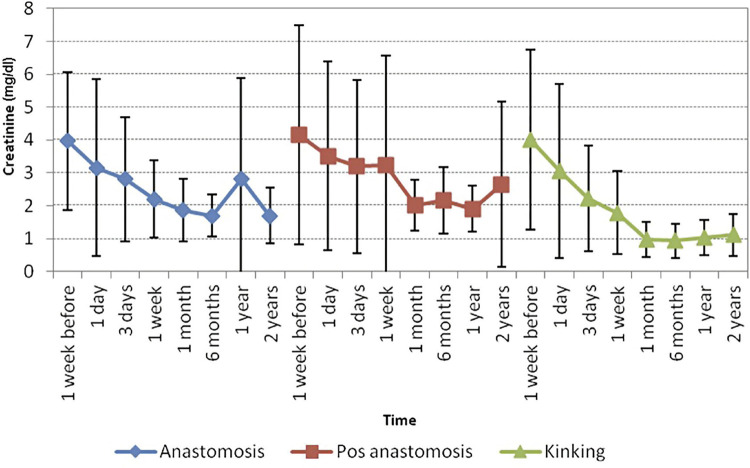
Comparison of creatinine progress regarding the site and cause of anastomosis. Data with mean+-Standard Deviation.

The cohorts were also similar in terms of Cr decrease (p=0.47), blood flow speed at doppler ultrasound (p=0.63), and prevalence of hypertension (p=0.11). Improvement of renal function showed a tendency to be better when due to kinking compared to other sites of stenosis. However this data was not statistically significant, probably due to the small sample size (Figure-4).

### Classical vs expanded donation criteria

Comparison regarding the outcomes of renal function, blood pressure behaviour and mean blood flow speed between patients with classical versus expanded donation criteria (>60 years-old, or donors between 50-59 years with 2 of 3 of the following criteria: hypertension; creatinine greater than 1.5mg/dL or creatinine clearance between 50-70mL/s before removal of the organ; and cause of death stroke), was performed. There was no statistically significant difference (p >0.05).

## DISCUSSION

TRAS remains the most frequent vascular complication in patients undergoing renal transplantation. It is responsible for 1 to 5% of cases with refractory hypertension following transplantation. The incidence of TRAS is extremely variable; it was reported an incidence of 2.3% in a large series of 2.013 kidney-transplanted patients (9); it was reported an increase in TRAS diagnosis, with the incidence rising from 2.4% to 12.4% since the routine introduction of diagnostic doppler ultrasonography (10); these results are similar to those of Mammen et al. (11), who reported a rise from 1.7% to 7.9%. The same group also reported that patients with TRAS treated with PTA/stenting had a 5-year survival rate of 76.3%, higher than kidney transplant patients who did not develop TRAS (72.9%), emphasising the effectiveness of the treatment. Although we performed doppler ultrassound routinelly after the renal transplant, our incidence of 0.9% is lower than the previously reported, possibly because we use a strict definition of imaging and clinical criteria instead of solely doppler characterization.

Early diagnosis and immediate therapeutic work-up plays an important role in the graft function. The angioplasty of renal artery stenosis has been an excellent therapeutic method in the management of these cases, since it presents few complications with significantly improvement in renal function and blood pressure control. This retrospective evaluation highlights the safety of endovascular management of TRAS and we could succesfully treat all cases without any complication. Furthermore, TAP’s efficiency was symbolized by renal function and graft preservation with significant reduction of mean blood pressure to normal parameters. After a two-year follow-up, no cases of restenosis occurred and these patients are being followed closely for an extended period.

TRAS is a serious complication of renal transplantation. There is a higher incidence of TRAS in the first 6 months of transplantation and in elderly transplanted patients. Early appearing stenosis are mainly due to traumatic intimal injury during recovering of the organ or vessel manipulation, kinking of the artery when it is longer than the vein, or technical problems with the vascular suture. Stenosis occurring later, sometimes in terms of years, reflect allograft renal artery hyperplasia or renal and/or iliac atherosclerotic evolution. Late and diffuse stenosis might also reflect endothelial damage related to immune response. In our series, the median time to presentation was 145 days and is consistent with the literature. We found cases of kinking to present in younger patients and with lower dyastolic blood pressure than in patients with anastomotic or post-anastomotic stenosis.

We realize the importance of both blood pressure and renal function normalization to improve endurance of the transplanted graft and survival of recipients. We could successfully normalize both parameters in all patients identified with TRAS who were treated with TAP within a 2-year follow-up, which is longer than some recent series (12-14). Our results are consistent with the literature (12-15) and corroborate the effectiveness of endovascular treatment of TRAS with PTA/stenting. Despite a reduction in blood pressure values, a reduction in the number of antihypertensive drugs was not observed. This is similar to the findings of a recent publication by Braga et al. (14) We attribute this fact to be a result of a closer and more strict outpatient follow-up with close monitoring of the blood pressure to assure normal levels at all times and a more precise pressure control.

This investigation has potential draw-backs that cannot be overlooked. It is a retrospective analysis and therefore suscetible to selection and measurement bias. However, our database is collected in a prospective manner and missing data is extremelly rare. Also, the concise group of urologists and nephrologists following those patients together minimize such biases. Another limitation is the small number of patients analyzed. The number of individuals with suspected TRAS is much larger, nevertheless we have a strict criteria to define TRAS which explain the low amount of patients identified with the disease at our Institution. In addition, most differences found in the investigation achieved significance even in this relatively short series. A larger prospective multi-institutional study could provide a better insight into the subgroup analysis.

Endovascular treatment with PTA/stenting is a safe and effective option for managing TRAS as it preserves vascular permeability in short and medium term, ensuring the functionality of the graft and normalization of blood pressure and renal function. In addition, patients with TRAS due to kinking showed a tendency of better results in terms of renal function improvement.
